# H_2_O_2_-mediated relaxation in a swine model of ischemic heart disease and exercise training: mechanistic insights and the role of Kv7 channels

**DOI:** 10.1007/s00395-025-01129-6

**Published:** 2025-07-12

**Authors:** T. S. Self, J. F. Bray, C. L. Heaps

**Affiliations:** 1 Department of Physiology & Pharmacology, MS4466,, College of Veterinary Medicine & Biomedical Sciences, Texas A&M University, College Station, TX 77843 USA; 2 Michael E. DeBakey Institute for Comparative Cardiovascular Science & Biomedical Devices, College of Veterinary Medicine & Biomedical Sciences, Texas A&M University, College Station, TX USA

**Keywords:** Microcirculation, Kv Channels, Hydrogen peroxide, Exercise training, Ischemic heart disease, Wire myography

## Abstract

We previously reported that H_2_O_2_-mediated arteriolar dilation impaired by chronic occlusion is corrected with exercise training and that BKCa and Kv channels both contribute to these adaptations. To gain additional understanding of the specific Kv channel isoforms influenced by ischemia and exercise, we hypothesized that the redox-sensitive Kv1, Kv2, and Kv7 channel subfamily isoforms would be the primary end effectors of this exercise-augmented channel contribution. Yucatan miniature swine were surgically instrumented with an ameroid occluder around the proximal left circumflex coronary artery, inducing an ischemic vascular bed, while arterioles fed by the left anterior descending artery served as nonoccluded, control vessels for each animal. Animals were randomly assigned to sedentary (normal pen activity) or exercise-trained (treadmill; 5 days/week; 14 weeks) groups. Kv channels were targeted, ex vivo, in wire myography and electrophysiology studies for functional analyses, while arteriolar lysates and isolated vascular smooth muscle cells were utilized for immunoblot and immunofluorescence. We show that coronary occlusion impairs Kv7 channel contribution to H_2_O_2_-mediated relaxation that is reversed with exercise training. Whole-cell voltage clamp recordings demonstrated no changes in XE991-sensitive currents among groups, and no significant differences in Kv7 channel protein were detected. Immunofluorescent analyses revealed a decrease in colocalization of PKA with Kv7.1 channels following occlusion and increased localization with both Kv7.1 and Kv7.5 channels following exercise training. Taken together, these studies demonstrate that Kv7 channel uncoupling from a prominent vasorelaxation signaling axis results from coronary occlusion and is restored following exercise training, highlighting this subfamily as a potential therapeutic target.

**Supplementary Information:**

The online version contains supplementary material available at 10.1007/s00395-025-01129-6.

## Highlights

- Arterioles distal to occlusion demonstrate reduced Kv7 channel sensitivity to H_2_O_2_
- Exercise-training recouples H2O2 and Kv7 channels for arteriolar relaxation
- Whole-cell voltage-clamp recordings suggest indirect Kv7 channel activation by H_2_O_2_
- PKA and Kv7 channel localization is altered by occlusion and exercise training

**Supplementary Information:**

The online version contains supplementary material available at 10.1007/s00395-025-01129-6.

## Introduction

In recent years, there has been a shift in the physiological paradigm regarding reactive oxygen species (ROS) in the coronary circulation and their role as pathogenic mediators [7, 24, 39]. While once regarded as promising therapeutic targets, meta-analyses of randomized clinical studies have shown that supplementation of nonspecific antioxidants are ineffective and often detrimental to diseased patients [2, 41]. Many studies have demonstrated ROS-mediated signaling axes that mitigate the negative effects of ischemia on the working myocardium [28, 29, 38, 60, 61]. However, our understanding of the specific contributions of numerous ROS candidates, such as H_2_O_2_, remains a topic of debate and high interest in experimental studies. In fact, under pathophysiological conditions, nitric oxide-induced arteriolar relaxation is often impaired, and EDHF-mediated relaxation is enhanced as a compensatory mechanism [1, 17]. Moreover, H_2_O_2_ is associated with this rescue of arteriolar relaxation in the ischemic myocardium [60]. Understanding the role of ROS-mediated signaling in the ischemic heart and identifying specific oxidant species is essential for designing customized treatment or intervention options for diseased patients.

Exercise training is well established as an effective, nonpharmacological therapeutic for patients with ischemic heart disease (IHD), inducing vascular adaptations that improve endothelial and smooth muscle function, increasing blood flow to the compromised myocardium [13, 31, 33, 53]. However, the underlying mechanisms that drive these adaptations, especially those involving ROS, have yet to be fully characterized. Our laboratory has previously reported that exercise training significantly increases agonist-stimulated H_2_O_2_ levels and that H_2_O_2_ is a significant mediator for an augmented endothelium-dependent signaling pathway for arteriolar dilation after exercise training [60]. Moreover, we have previously demonstrated that both large-conductance Ca^2+^-activated K^+^ (BKCa) channels and voltage-gated K^+^ (Kv) channels contribute to H_2_O_2_-mediated vasodilation after endurance exercise training [28]. Thus, we sought to identify the specific Kv channel isoforms responsible for augmented H_2_O_2_-mediated vasoreactivity with subsequent analyses of second messenger signaling that may be driving these adaptations.

Kv channels populate the plasma membrane of vascular smooth muscle cells and present as significant end effectors for many dilatory pathways [12, 26, 57]. Although the Kv channel family constitutes at least 44 unique isoforms categorized into 12 subfamilies, Kv1, Kv2, and Kv7 have been identified as particularly important in the coronary circulation [26]. Interestingly, members of these families demonstrate redox-sensitivity [14, 15, 43, 45] and have been implicated as significant players for the regulation of vascular tone in health and disease in a variety of tissue beds. However, reports of Kv channel involvement in the regulation of coronary vascular tone are often limited to large coronary arteries and even fewer investigate their modulation by exercise. It is known that Kv channels play a significant role in regulating vascular smooth muscle membrane potential and are often modulated in health and disease. However, little is known about which specific isoforms drive these adaptations. We aimed to identify candidate Kv channel isoforms and their involvement in vascular adaptations of the coronary microcirculation associated with chronic coronary occlusion and exercise training in a swine model. We sought to interrogate redox-sensitive Kv channel involvement in H_2_O_2_-mediated relaxation, identify these channels contributions to whole-cell K^+^ current, and investigate protein levels and cellular colocalization of channel isoforms with protein kinase A (PKA), which is known to associate with K^+^ channels in other vascular beds [4].

Based on our previous studies and the Kv channel literature, the current studies were designed to test the hypothesis that chronic coronary artery occlusion would impair H_2_O_2_-mediated relaxation in the coronary microcirculation and that a progressive exercise-training regimen would restore this signaling pathway through increased an increased role of redox-sensitive Kv channels that were lost with persistent ischemic insult. We are the first to investigate the Kv7 channel subfamily in a swine model of IHD paired with exercise training as a therapeutic intervention. Using this clinically relevant and translational large animal model, we highlight a potential novel therapeutic target involved in the development of IHD and the recovery of vascular reactivity following exercise training.

### Materials and methods

#### Animals and surgical instrumentation

All animal protocols were approved by the Texas A&M University Institutional Animal Care and Use Committee and conformed to the National Institutes of Health (NIH) Guide for Care and Use of Laboratory Animals, 8th edition. Adult, female Yucatan miniature swine (6–7 months of age) were surgically instrumented with an ameroid occluder around the proximal left circumflex coronary artery (LCX), leaving the left anterior descending coronary artery (LAD) unobstructed, as routinely performed in our laboratory [20, 28, 51, 61]. Animals were pre-anesthetized by intramuscular injection with glycopyrrolate (anticholinergic; 0.004 mg/kg) and midazolam (benzodiazepine; 0.5 mg/kg), followed by ketamine (dissociative anesthetic; 20 mg/kg). Animals were masked, and general anesthesia was induced by 3% isoflurane. Animals were then intubated, and anesthesia was maintained with 2–3% isoflurane supplemented with O_2_ (1.5–1.75 L/min) during aseptic surgery and ventilated at a rate of 10–12 breaths per minute while monitoring heart rate by ECG, end-tidal CO_2_, and SPO_2_. Prior to surgery, animals were administered ceftiofur by intramuscular injection (Excede; antibiotic; 5.0 mg/kg) and intravenous injection of buprenorphine hydrochloride (Buprenex; analgesic; 0.02 mg/kg) and carprofen (non-steroidal anti-inflammatory; 4.4 mg/kg). Bupivacaine (local anesthetic) was injected intramuscularly in the 3rd, 4th, and 5th intercostal spaces (1 cc per space) prior to accessing the chest cavity. The neuromuscular blocker, pancuronium (0.1 mg/kg), was administered intravenously prior to incising the intercostal layers for accessing the chest cavity by Finochietto retractors. Following access to and evaluation of the proximal LCX artery, an ameroid occluder (2.5–3.5 mm) was placed to ensure a tight but nonconstructive fit. Bupivacaine was applied topically along the suture line (2 cc), during closure of the chest cavity. During surgery, animals were provided the following drugs as necessary: atropine (muscarinic receptor antagonist; 0.5 mg/kg), epinephrine (adrenergic receptor agonist; 10 µg/kg), and lidocaine (antiarrhythmic; 1 mg/kg). Buprenorphine hydrochloride (0.02 mg/kg) was administered every 3–6 h postoperatively, as needed for pain. Occlusion is gradual with complete occlusion achieved 2–3 weeks postoperatively [59]. Sedentary or progressive exercise-training protocols began 8 weeks postoperatively.

#### Sedentary and exercise-training protocols

Animals were randomly assigned to either a sedentary or exercise-training group. Sedentary animals were limited to normal pen activity. Exercise training consisted of a 14-week (5 days/week) progressive training regimen on a treadmill, beginning with a 5-min warm up (2–2.5 mph) followed by an endurance run (10–60 min; 3–5 mph) and 5-min cool down (2–2.5 mph). The endurance run began at 3 mph for 10–14 min during week 1 gradually increasing to 60 min at 5 mph by week 12. A short speed run was introduced at week 2 and maintained through the remainder of the protocol. The speed run began at 3.5 mph for 1 min gradually increasing to 6 mph for 8 min by week 12. Treadmill grade was maintained at 0% for the duration of the training protocol. Effectiveness of the exercise-training regimen was assessed by skeletal muscle citrate synthase activity in the medial and lateral heads of the triceps brachii muscle and by comparison of heart-to-body weight ratios. Animals were fed once daily. Exercise-trained animals were fed immediately after each exercise bout as positive reinforcement. All animals were provided water ad libitum.

#### Isolation of coronary arterioles

**Fig. 1:**
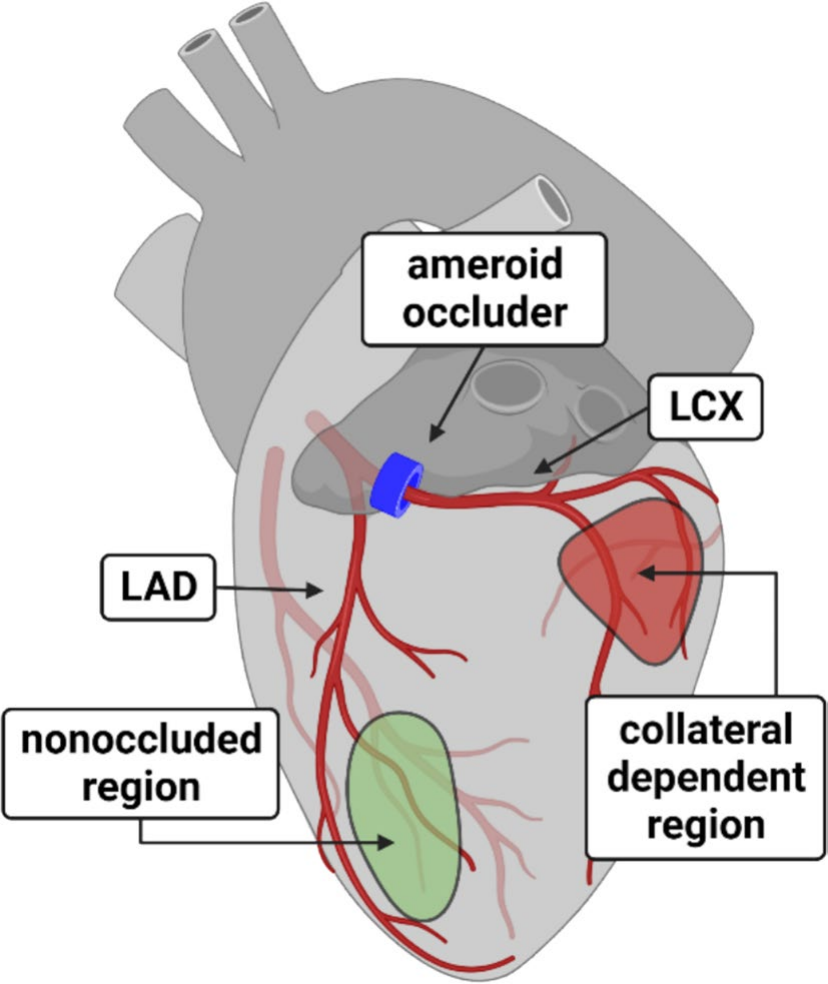
Model schematic of nonoccluded and collateral-dependent regions. Surgical instrumentation of an ameroid occluder on the proximal left circumflex coronary artery progressively induced a downstream ischemic vascular bed with complete occlusion typically occurring between 2–3 weeks postoperatively [48]. The left anterior descending coronary artery remained unobstructed, and the myocardial tissue supplied by this artery served as a nonoccluded control region. The shaded areas represent the two areas from which arterioles were isolated. *Created with biorender.com*

After the 14-week treatment protocol, animals were pre-anesthetized, intramuscularly, with xylazine (sedative; 2.25 mg/kg) and ketamine (35 mg/kg), 24–36 h after the last exercise bout. Animals were masked and anesthesia induced by 3% isoflurane. Animals were then intubated, and 3% isoflurane supplemented with O_2_ (1.5–1.75 L/min) was provided to maintain a surgical plane of anesthesia. Hearts were excised by left lateral thoracotomy and immediately placed in ice-cold (0–4 °C) Krebs bicarbonate buffer. Hearts were weighed for heart-to-body weight ratio comparisons. The ameroid occluder was viewed under a stereomicroscope to visually confirm complete occlusion of the LCX artery. Arterioles (~ 125 µm internal diameter; ~ 2 mm in length) were dissected free from surrounding tissue from both nonoccluded and collateral-dependent vascular beds supplied by the LAD and LCX coronary arteries, respectively. Arterioles were taken from the central region of the LAD and LCX supplied vascular beds (Fig. 1) to avoid heterogenous border zones [22, 47, 63]. All tissues were maintained in ice-cold (0–4 °C) Krebs bicarbonate buffer prior to experiments.

#### Isometric tension wire myography

Functional analyses of arteriolar reactivity were performed using specialized micro vessel myograph chambers (Danish Myo Technology). Isolated coronary arterioles were size matched for lumen diameter, cut to 1.5 mm axial length, and mounted for isometric tension recordings using 25 µm tungsten wires. Arteriole inner diameter, outer diameter, wall thickness, and axial length were measured using a calibrated micrometer eyepiece. Briefly, under view of an Olympus SZX7 stereo microscope, two tungsten wires (Danish Myo Technology) were carefully threaded through the vessel lumen and secured on the mounting feet of the myograph chamber: one attached to a force transducer, and the other to a micrometer. Initially, the micrometer was set at the point of zero tension. Vessels were continuously bathed in aerated (95% O_2_ balanced with 5% CO_2_) Krebs bicarbonate buffer, and warmed to 37 °C. After mounting, vessels were allowed to equilibrate for 1 h then subjected to a passive length–tension normalization. The passive length–tension relationship was generated for each vessel to determine the optimal length for subsequent studies. Vessel viability was tested by 3 exposures to high KCl (60 mM) with Krebs bicarbonate washes in between exposures before a final 15-min equilibration period. Vessel constriction was achieved by application of endothelin-1 (ET1; Tocris; 2 nM). Upon stable constriction, increasing concentrations of H_2_O_2_ (1e-9 to 1e-3 M) in whole-log steps or the Kv7 channel activator, ML213 (1e-9 to 1e-5 M), in half-log steps, were applied to obtain concentration–response curves and evaluate vessel reactivity. The relaxation response was allowed to stabilize prior to application of the next consecutive concentration. When selective pharmacological antagonists were used, incubation was permitted for 15 min prior to constriction with ET1 and remained in contact with the tissues for the duration of the experiment. Drug vehicle constituted < 0.1% of the myograph tissue bath. Following all concentration–response curves, the chamber bath was replaced with 0Ca^2+^/high Mg^2+^ Krebs bicarbonate buffer with sodium nitroprusside (SNP; Sigma; 0.1 mM) to achieve maximal relaxation for subsequent analyses. Basal tone is a physiological parameter calculated from the tension at optimal stretch (the pressure that corresponds with optimal contractility) minus the tension when the vessel is exposed to Ca^2+^-free solution and sodium nitroprusside (100 µM). Data were recorded using PowerLabs signal amplifier (AD Instruments) and Labchart 7 acquisition software (AD Instruments) and stored on a local drive.

#### Smooth muscle cell isolation

Freshly isolated coronary arterioles (~ 125 µm internal diameter) from either the nonoccluded or collateral-dependent vascular regions were digested in 0.5 mL low-Ca^2+^ (0.5 mM) physiological buffer containing 1.4 mg/mL papain, 0.4 mg/mL DTT, 0.4 mg/mL bovine serum albumin, and 2 mg/mL collagenase II for 30–45 min at 37 °C. Following incubation, vessels were dispersed by gentle trituration, and then centrifuged at 1000 rpm for 30 s. The supernatant was removed and discarded, and then the cell pellet resuspended with enzyme-free low-Ca^2+^ physiological buffer. Centrifugation and resuspension were repeated two times to ensure an enzyme-free environment. Smooth muscle cells are morphologically distinguishable from other cell types such as endothelial cells and fibroblasts [6]. Isolated cells were kept in low-Ca^2+^ solution at 0–4 °C for subsequent experiments (0–6 h).

#### Whole-cell voltage clamp

K^+^ currents were recorded from isolated arteriolar smooth muscle cells (SMCs) using standard whole-cell voltage clamp techniques as routinely performed in our laboratory [11, 21, 61]. Current recordings were performed under physiological K^+^ concentrations (140 mM intracellular; 5 mM extracellular). Cells were continuously superfused under gravity-driven flow at room temperature (22–25 °C) with physiological saline solution containing (in mM): 138 NaCl, 5 KCl, 2 CaCl_2_, 1 MgCl_2_, 10 HEPES, and 10 glucose; pH 7.4 with NaOH. Heat-polished glass pipettes (2–5 MΩ) were used to achieve the GΩ seal and backfilled with solution containing (in mM): 120 K-glutamate, 20 KCl, 10 EGTA, and 10 HEPES; pH 7.2 with KOH. EGTA was used to chelate intracellular Ca^2+^ to minimize the contribution of BKCa channels. Ionic currents were amplified by an Axopatch 200B path-clamp amplifier (Molecular Devices). Currents were stimulated by 500 ms step depolarizations ranging from − 70 mV to + 60 mV in 10 mV increments from a holding potential of − 80 mV. Currents were low pass filtered with a cutoff frequency of 1.0 kHz, digitized at 2.5 kHz, and stored on a local hard drive for subsequent analyses. Leak subtraction was not performed. When pharmacologic agents were used, incubation was allowed until K^+^ currents at + 20 mV stabilized (2–3 min). Activation curves were calculated from channel conductance at each voltage step for each cell and fitted to a Boltzmann distribution. From channel conductance, half-maximal activation (V_1/2_) and slope were calculated in which V_1/2_ is the test potential at which conductance is half-maximal and slope reflects the voltage dependence of channel activation. Data acquisition and analyses were performed using pClamp 9 software (Molecular Devices).

#### qPCR analyses

Freshly isolated coronary arterioles (~ 125 µm internal diameter) from abattoir swine were collected and snap frozen in liquid N_2_ for qPCR analyses. Total RNA extraction began by grinding frozen vessels with a mortar and pestle and adding 200 µL Trizol. Following a 20-min incubation at room temperature, 50 µL chloroform was added, samples were centrifuged for 2 min at 14,000 rpm, supernatants collected, and 40 µg glycogen, 10% 3 M NaAcetate, and an equal volume of 5 M LiCl/80% isopropanol alcohol[32] was added and incubated for 15 min at − 80 °C. Samples were then centrifuged at 14,000 rpm and supernatants collected. Pellets were washed with −  20 °C 80% ethanol and resuspended in nuclease-free water. RNA concentrations were determined by Abs260, nanodrop UV spectrophotometry. cDNA synthesis reactions included 1 µg of total RNA with 1 × iScript Adv cDNA mic (BioRad 172-5037) in 20 µL reactions. Real-time PCR reactions were performed using a BioRad CFX96 thermocycler with DDo Adv SYBrGreen supermix (BioRad 172-5271), 25 ng cDNA, and 1 pmol/µL of Kv7 channel isoform primers sourced from IDT DNA. Primer sets are outlined in Table 1. Cycling parameters were as follows: Kv7.1 and Kv7.2: 5eC/:10, 72C/:20, 95C/:10 × 40 cycles; Kv7.3, Kv7.4, and Kv7.5: 62C/:20, 95C/:10 × 40 cycles. Positive RNA controls for Kv7.1, Kv7.2, and Kv7.3 were extracted from swine brain tissue. Positive RNA controls for Kv7.4 and Kv7.5 were extracted from swine myocardium. n values for Kv7 isoforms are as follows: Kv7.1 *n* = 7, Kv7.2 *n* = 4, Kv7.3 *n* = 4, Kv7.4 *n* = 14, and Kv7.5 *n* = 11. Data were analyzed using the ΔΔCT method with β-actin as the reference gene and expressed relative to the lowest expressed isoform. PCR products were verified by sequencing (Eton Bioscience; San Diego, CA).

**Table 1:** Primers for Kv7 isoforms

Isoform	Primer
Kv7.1	F: 5’ GTCCACCATTGAGCAGTATGT 3’ R: 5’ TACTCCGTCCCGAAGAACA 3’
Kv7.2	F: 5’ CACCATCAAGGAGTACGAGAAG 3’ R: 5’ CCCAGATCCGCACAAAGT 3’
Kv7.3	F: 5’ GCAGCCACCTTCTCCTT AT 3’ R: 5’ CTCCTCTTCTCAAAGTGCTTCT 3’
Kv7.4	F: 5’ ACTCACGGTGGACGATGTTAT 3’ R: 5’ CAGCGTCTCCTTGAATTTCCTT 3’
Kv7.5	F: 5’ CTGTTGCCGATATAGAGGATGG 3’ R: 5’ CAACTGCAATCGAAGCGATAAG 3’
β-actin	F: 5’ AAGATCAAGATCATCGCGCCTCCA 3’ R: 5’ ACTCCTGCTTGCTGATCCACATCT 3’

Primer sequence for Kv7 isoforms and β-actin

#### Immunoblots

Freshly isolated coronary arterioles (~ 125 µm internal diameter, 8–10 arterioles per region per animal) were snap frozen in liquid N_2_ and stored at -80 °C until subsequent immunoblot analyses. Lysate (20 µg) was prepared from whole arterioles and subjected to SDS–polyacrylamide gel electrophoresis (4–20% gradient gel), transferred to a PVDF membrane, blocked with 5% non-fat dehydrated milk reconstituted in Tris-buffered saline with tween (TTBS), and then probed overnight with primary antibody in TTBS. Primary antibodies included: Kv7.1 (Boster A00310-1; 1:1000), Kv7.4 (LS Bio B13617; 1:1000), Kv7.5 (LS Bio C405104; 1:1000), and GAPDH (Advanced immunochemical no. RGM2-200; 1:5000). After washing in TTBS, membranes were incubated in the appropriate horseradish peroxidase-conjugated species-specific anti-IgG. Scanning densitometry (Fujifilm LAS-3000 imager) was used to quantify signal intensities which were then normalized to GAPDH. Preliminary experiments were performed to confirm primary antibody specificity (data not shown).

#### Immunolabeling of freshly isolated smooth muscle cells

Freshly isolated arteriolar SMCs were plated onto glass slides coated in Cell-Tak (Corning; Cat. no. 354240) and incubated at 37 °C for 10 min under either control, non-treated (5 mM Krebs) or H_2_O_2_-treated (50 µM H_2_O_2_ added to 5 mM Krebs) conditions, desiccated, and fixed with paraformaldehyde (Thermo Fisher Scientific; Cat. No. 157-8) diluted to 2% in PBS (pH: 7.4; 10 min at 25 °C). Slides were then rinsed in PBS and incubated in glycine (100 mM) for 10 min. Three PBS washes (3 min each) concluded fixation. Slides were incubated in primary antibodies (Kv7.1: Boster A00310-1, Kv7.4: LS Bio B13617, Kv7.5: LS Bio C405104, PKA-R1: BD Biosciences 610165), diluted to 1:100 in 1X SSC buffer (150 mM NaCl, 15 mM Na Citrate, pH 7.4) with 0.1% Triton X-100 and 1% BSA (Sigma; Cat. No. A7030) overnight at 4 °C. The following morning, slides were rinsed 3 times in PBS then incubated in secondary antibodies (anti-rabbit Alexa 488 and anti-mouse Alexa 647), and nuclear stain (Hoechst; Enzo 33258) diluted to 1:500 in 1X SSC buffer with BSA and Triton X-100 for 1 h at 25 °C. Subsequently, slides were rinsed in PBS 3 times, covered in ProLong anti-fade solution (Thermo Fisher Molecular Probes; Cat. No. P36934) and topped with a glass coverslip. Preliminary experiments determined the optimal dilutions for all antibodies. Control experiments include autofluorescence of non-incubated cells, incubation in primary only, and incubation in secondary only (data not shown).

#### Laser-scanning confocal microscopy

Fluorescent images of isolated arteriolar smooth muscle cells, labeled for Kv7 isoforms and PKA were acquired on a Zeiss LSM 780 / Airy Scan NLO Multiphoton Microscope using a Plan-Apochromat 63x/1.40 Oil DIC M27 objective. Pinhole size was set to 1 AU. Imaging fields were optimized, and samples illuminated with 633, 488, and 405 nm diode lasers. 1912 × 1912 pixel images were acquired at 71 nm/pixel resolution. Emission bandpass detection was as follows: Hoechst: 415–481 nm, AlexaFluor488: 490–561 nm, and AlexaFluor647: airyscan. All hardware and software parameters were maintained identically within experiments.

#### Data analyses

Animal body weight, heart-to-body weight ratio, and citrate synthase activity assays were compared between sedentary and exercise-trained groups by Student’s t tests. Coronary arteriole dimensions and characteristics were compared by one-way ANOVA. Concentration–response curves are presented as percent decrease in tension relative to the maximal relaxation to account for variability in initial and passive diameters between arterioles. The following equation was used to calculate percent relaxation at each concentration of vasodilator: [(T-T_0_)/(T_0_-T_min_)] × 100 where T is the stabilized tone after each concentration step, T_0_ is the stable tone when preconstricted with ET1 (2 nM), and T_min_ is the minimal tension after treatment with 0Ca^2+^/high Mg^2+^ Krebs bicarbonate buffer solution and SNP (0.1 mM). Concentration–response relationships are presented as means ± standard error of the mean (SEM). Concentration–response relationships were analyzed by two-way ANOVA with repeated measures (RM) followed by Bonferroni correction for multiple comparisons when main effects were identified. EC_50_ values are representative of the concentration of H_2_O_2_ or ML213 that produced 50% of the maximal relaxation in each arteriole. EC_50_ values are reported as the means ± SEM and were analyzed by either Student’s t test, one-way ANOVA followed by Bonferroni post hoc analysis when appropriate, or Kruskal–Wallis nonparametric test when data were not normally distributed. Basal tone and developed tension in response to drug incubation were analyzed by one-way ANOVA or Kruskal–Wallis nonparametric test when data were not normally distributed. Protein analyses by immunoblot are reported as means ± SEM and were compared by one-way ANOVA. Protein colocalization by Pearson’s correlation coefficient (PCC) analyses are presented as mean ± SEM and were analyzed by independent samples Kruskal–Wallis test with Bonferroni correction for multiple comparisons. Current–voltage relationships and activation curves were analyzed by two-way RM ANOVA, while V_1/2_ and slope were analyzed by ANOVA on ranks. Animal values (n) are reported in parenthesis as (n = animal number) except for electrophysiology experiments and immunofluorescence being reported as (n = animal number, total cell number) with each animal being represented by 3–4 cells for voltage clamp and 10 cells for immunofluorescence. A p value ≤ 0.05 was considered significant.

## Results

### Efficacy of exercise training

The effectiveness of the 14-week exercise-training regimen was confirmed by comparing heart weight-to-body weight ratios and skeletal muscle oxidative enzyme capacity of exercise-trained compared with sedentary swine. Body weight, measured at the time of termination, did not differ between the groups (*p* = 0.55), but the heart weight-to-body weight ratio was significantly increased after completion of the exercise-training regimen (*p* < 0.001) and reported in Table 2. Further, citrate synthase activity was significantly increased in the triceps lateral head (TLtH) and triceps medial head (TMH) skeletal muscles in the forelimbs of exercised-trained compared to sedentary swine (*p* = 0.002 and *p* = 0.003, respectively).

**Table 2:** Efficacy of the exercise training program

	*n*	BW (kg)	HW/BW (g/kg)	CS activity (µmol/min/g)	
				TMH	TLtH
Sedentary	25	34.7 ± 1.1	4.7 ± 0.1	41.4 ± 2.1	40.3 ± 1.0
Exercise trained	25	35.5 ± 0.8	5.4 ± 0.1*	50.7 ± 2.0*	46.5 ± 1.6*

Values are represented as means ± SEM. *n*, number of animals. *BW* body weight. *HW/BW* heart weight-to-body weight ratio. *CS* citrate synthase. *TMH* triceps medial head. *TLtH* triceps lateral head. *p < 0.05 vs. sedentary

### Coronary arteriole characteristics

Table 3 provides dimensional properties of the isolated arterioles utilized for tension wire myography. No significant differences in vessel dimensions or characteristics were observed between nonoccluded or collateral-dependent arterioles of sedentary or exercise-trained swine (Table 3).

**Table 3:** Coronary arteriole dimensions and characteristics

	*n*	OD (µm)	ID (µm)	WT (µm)	AL (mm)	BT (mN/mm)
Sedentary						
Nonoccluded	14	246 ± 11	135 ± 7	54 ± 3	1.45 ± 0.03	0.26 ± 0.05
Collateral dependent	10	252 ± 11	128 ± 8	61 ± 4	1.43 ± 0.04	0.33 ± 0.07
Exercise trained						
Nonoccluded	10	214 ± 13	113 ± 10	51 ± 2	1.43 ± 0.03	0.24 ± 0.03
Collateral dependent	11	243 ± 17	135 ± 12	54 ± 4	1.50 ± 0.04	0.22 ± 0.04

Values are represented as means ± SEM. *n*, number of arterioles (1 per animal). *OD* outer diameter. *ID* inner/luminal diameter. *WT* wall thickness. *AL* axial length. *BT* basal tone after passive length–tension normalization, prior to ET1 constriction. No significant differences found

### Effect of occlusion and exercise training on H_2_O_2_-mediated relaxation

**Fig. 2:**
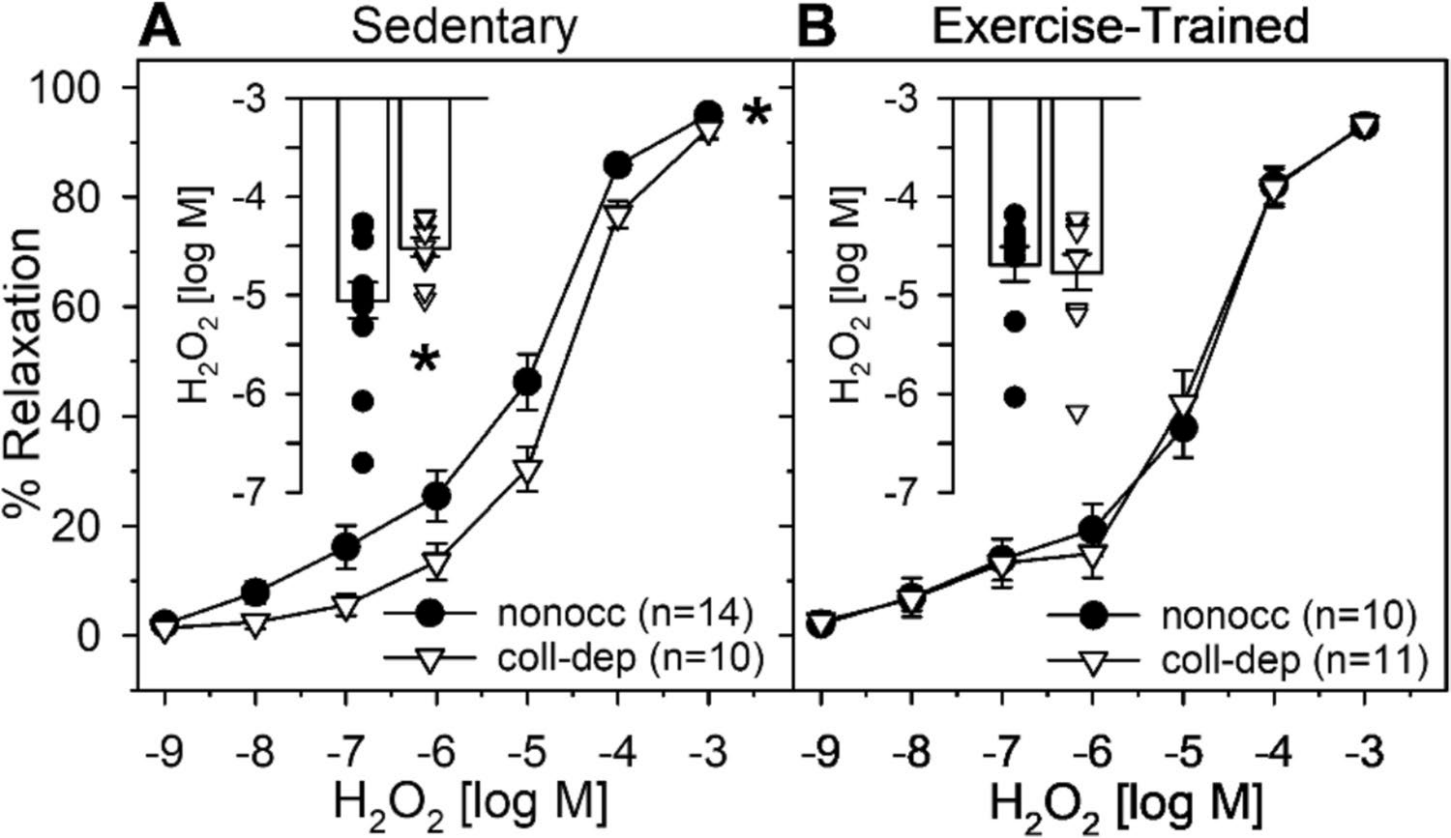
*Effect of chronic coronary artery occlusion and exercise training on H*_*2*_*O*_*2*_*-mediated relaxation.* **A**: Chronic coronary artery occlusion significantly impaired H_2_O_2_-mediated relaxation compared to nonoccluded arterioles (*p* = 0.013). EC_50_ values (inset bar graph) revealed reduced sensitivity to H_2_O_2_ in collateral-dependent compared to nonoccluded arterioles (*p* = 0.030). **B**: Following exercise training, concentration–response curves (*p* = 0.968) and EC_50_ values (*p* = 0.763) demonstrated no significant differences between nonoccluded and collateral-dependent arterioles. *p < 0.05. Values represented as means ± SEM

We evaluated the effect of chronic coronary occlusion and exercise training on H_2_O_2_-mediated relaxation of coronary arterioles using isometric tension wire myography. Analyses revealed that H_2_O_2_-mediated relaxation was significantly impaired in collateral-dependent compared to nonoccluded arterioles of sedentary animals, indicated by the significant rightward shift in the H_2_O_2_ concentration–response curve (Fig. 2A; *p* = 0.013). Further, the sensitivity to H_2_O_2_, represented by the EC_50_ value, was significantly decreased in collateral-dependent arterioles compared with arterioles from the nonoccluded region (Fig. 2A inset; *p* = 0.030). In contrast, after exercise training, no significant difference in the H_2_O_2_ concentration–response curves (Fig. 2B; *p* = 0.968) or EC_50_ values (Fig. 2B inset; *p* = 0.763) was detected between collateral-dependent and nonoccluded arterioles.

### Kv channel contribution to H_2_O_2_-mediated relaxation

**Fig. 3:**
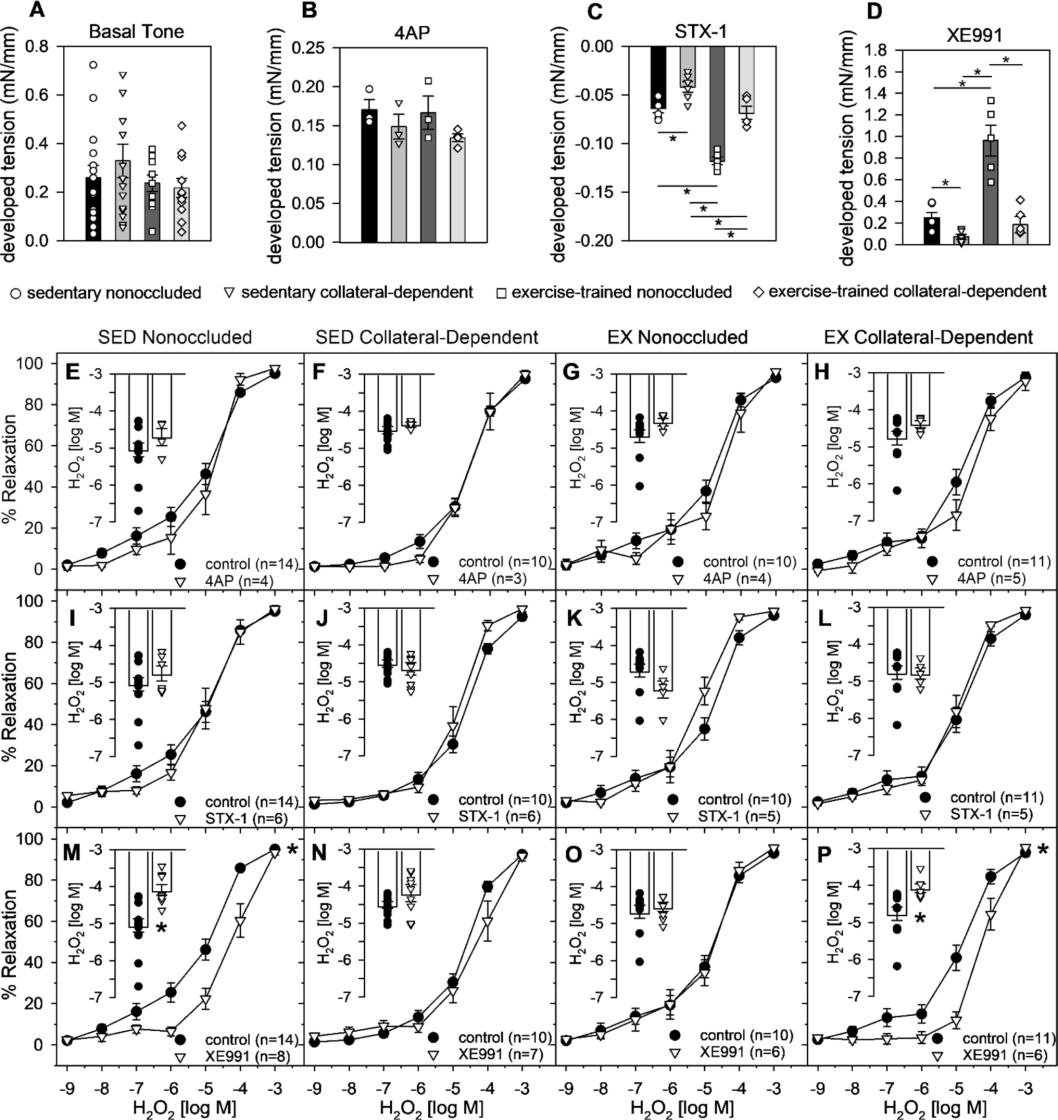
*Contribution of candidate Kv channels to H*_*2*_*O*_*2*_*-mediated relaxation following chronic coronary artery occlusion and exercise training.* **A**: Arteriolar basal tone was unchanged by occlusion or exercise training (*p* = 0.482). **B**: Developed tension following incubation in 4AP was unchanged by occlusion or exercise training (*p* = 0.263). **C**: Relaxation following incubation in stomatoxin-1 was significantly impaired by occlusion (*p* = 0.038) and increased by exercise training (*p* = 0.012). **D**: Developed tension following incubation in XE991 was significantly reduced by occlusion (*p* = 0.045) and recovered by exercise training. **E**–**H**: 4-Aminopyridine (4AP; 3 mM) did not significantly modify H_2_O_2_-mediated relaxation in any treatment group. **I**-**L**: Stromatoxin-1 (STX-1; 100 nM) did not significantly modify H_2_O_2_-mediated relaxation in any treatment group. **M**: XE991 (10 µM) significantly rightward shifted the H_2_O_2_ concentration–response curve (p = 0.003) and attenuated H_2_O_2_ sensitivity as determined by EC50 measures (inset; p = 0.002) of nonoccluded arterioles from sedentary animals (*p* = 0.003). **N**: XE991 did not significantly modify the H_2_O_2_ concentration–response curve (*p* = 0.234) nor H_2_O_2_ sensitivity (inset; p = 0.247) of collateral-dependent arterioles from sedentary animals. **O**: XE991 did not significantly modify the H_2_O_2_ concentration–response curve (*p* = 0.989) and H_2_O_2_ sensitivity (inset; p = 0.851) of nonoccluded arterioles from exercise-trained animals. **P**: XE991 significantly rightward shifted the H_2_O_2_ concentration–response curve (*p* = 0.012) and attenuated H_2_O_2_ sensitivity (inset; p = 0.021) of collateral-dependent arterioles from exercise-trained animals. *p < 0.05. Values represented as means ± SEM

We examined the contribution of candidate, redox-sensitive, Kv channels (Kv1, Kv2, and Kv7 subfamilies) to H_2_O_2_-mediated relaxation in the coronary microcirculation before and after occlusion and exercise training. Basal tone of isolated arterioles was unchanged by occlusion or exercise training (Fig. 3A; *p* = 0.482) and developed tension in response to incubation with 4-aminopyridine (4AP; 3 mM) was unchanged by occlusion or exercise training (Fig. 3B; *p* = 0.263). However, incubation with STX-1 (100 nM) reduced basal tone in all arterioles (Fig. 3C); an effect that was significantly diminished by chronic coronary occlusion (*p* = 0.038; sedentary nonoccluded vs. collateral-dependent) and restored by exercise training (*p* = 0.012; collateral-dependent sedentary vs. exercise-trained). Incubation with XE991 resulted in developed tension that was reduced by chronic coronary occlusion (Fig. 3D; *p* = 0.045; sedentary nonoccluded vs. collateral-dependent) and recovered by exercise training (*p* = 0.653; sedentary nonoccluded vs. exercise-trained collateral-dependent). Furthermore, basal tone following incubation in XE991 (10 μM) was significantly increased by exercise training in nonoccluded arterioles (*p* = 0.046). The Kv channel inhibitor, 4AP (3 mM), failed to alter H_2_O_2_-mediated relaxation, as evaluated by both concentration–response curves and H_2_O_2_ sensitivity (EC50 values; inset graph), in sedentary nonoccluded (Fig. 3E; *p* = 0.452; inset, *p* = 0.598), sedentary collateral-dependent (Fig. 3F; *p* = 0.921; inset, p = 0.947), exercise-trained nonoccluded (Fig. 3G; *p* = 0.523; inset, *p* = 0.221), and exercise-trained collateral-dependent (Fig. 3H; *p* = 0.197; inset, *p* = 0.214) arterioles. The selective, Kv2.1 inhibitor, stromatoxin-1 (STX-1; 100 nM), failed to modify H_2_O_2_-mediated relaxation in sedentary nonoccluded (Fig. 3I; *p* = 0.665; inset, *p* = 0.375), sedentary collateral-dependent (Fig. 3J; *p* = 0.149; inset, *p* = 0.453), exercise-trained nonoccluded (Fig. 3K; *p* = 0.386; inset, *p* = 0.112), and exercise-trained collateral-dependent (Fig. 3L; *p* = 0.844; inset, *p* = 0.909) arterioles. In contrast, the Kv7 subfamily inhibitor, XE991 (10 µM), significantly attenuated H_2_O_2_-mediated relaxation in sedentary nonoccluded arterioles (Fig. 3M; *p* = 0.003; inset, *p* = 0.002); this effect was lost following chronic coronary artery occlusion (Fig. 3N; *p* = 0.234; inset, *p* = 0.180). Interestingly, exercise training restored the contribution of Kv7 channel isoforms to H_2_O_2_-mediated relaxation in arterioles distal to coronary occlusion, indicated by the significant attenuation of H_2_O_2_-mediated relaxation following incubation with XE991 (Fig. 3P; *p* = 0.012; inset, *p* = 0.021). Surprisingly, XE991 failed to inhibit the H_2_O_2_ concentration–response curve in nonoccluded arterioles following exercise training (Fig. 3O; *p* = 0.989; inset, *p* = 0.851).

### Kv7 channel activation by ML213

**Fig. 4:**
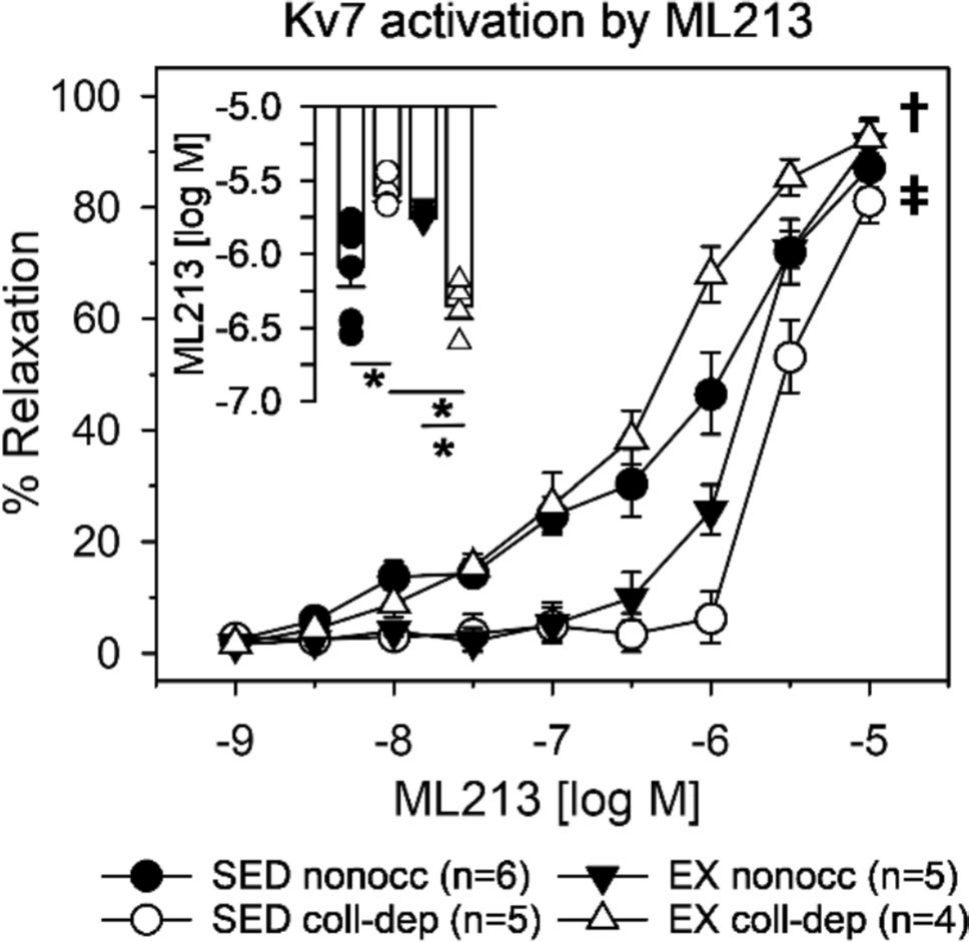
*Differential activation of Kv7 channels by ML213 before and after chronic coronary occlusion and exercise training.* Kv7 channels were significantly less responsive to activation by ML213 in collateral-dependent arterioles than those from the nonoccluded region of sedentary animals (*p* = 0.006). Further, the sensitivity to ML213 in collateral-dependent arterioles of sedentary animals was significantly reduced (inset; *p* = 0.002). Exercise training restored Kv7 activation by ML213 in the collateral-dependent region (*p* = 0.001), while nonoccluded arterioles tended to be less responsive, although not statistically, compared to the sedentary nonoccluded region (*p* = 0.159). Moreover, the ML213 sensitivity was increased in the collateral-dependent region (inset; *p* < 0.001) and decreased, although not statistically, in the nonoccluded region (*p* = 0.118) of exercise-trained animals compared to sedentary counterparts. *p < 0.05; †*p* < 0.05 sedentary collateral-dependent vs. sedentary nonoccluded; ‡*p* < 0.05 exercise-trained collateral-dependent vs. sedentary collateral-dependent. Values represented as means ± SEM

We challenged coronary arterioles from nonoccluded and collateral-dependent regions of sedentary and exercise-trained animals with increasing concentrations of the Kv7 channel activator, ML213 (1e-9–1e-5). Chronic coronary artery occlusion significantly attenuated the concentration–response curve in sedentary animals (Fig. 4; *p* = 0.006), and significantly reduced the sensitivity of arterioles to ML213 (Fig. 4; inset, *p* = 0.002). Interestingly, exercise training restored Kv7 channel activation by ML213 in collateral-dependent arterioles (Fig. 4; *p* = 0.001) and increased sensitivity to ML213 (Fig. 4; inset, *p* < 0.001). Nonoccluded arterioles of exercise-trained animals tended to be less sensitive to ML213 compared with those of sedentary swine, although not statistically (*p* = 0.159).

### Kv7 channel expression and protein content

**Fig. 5:**
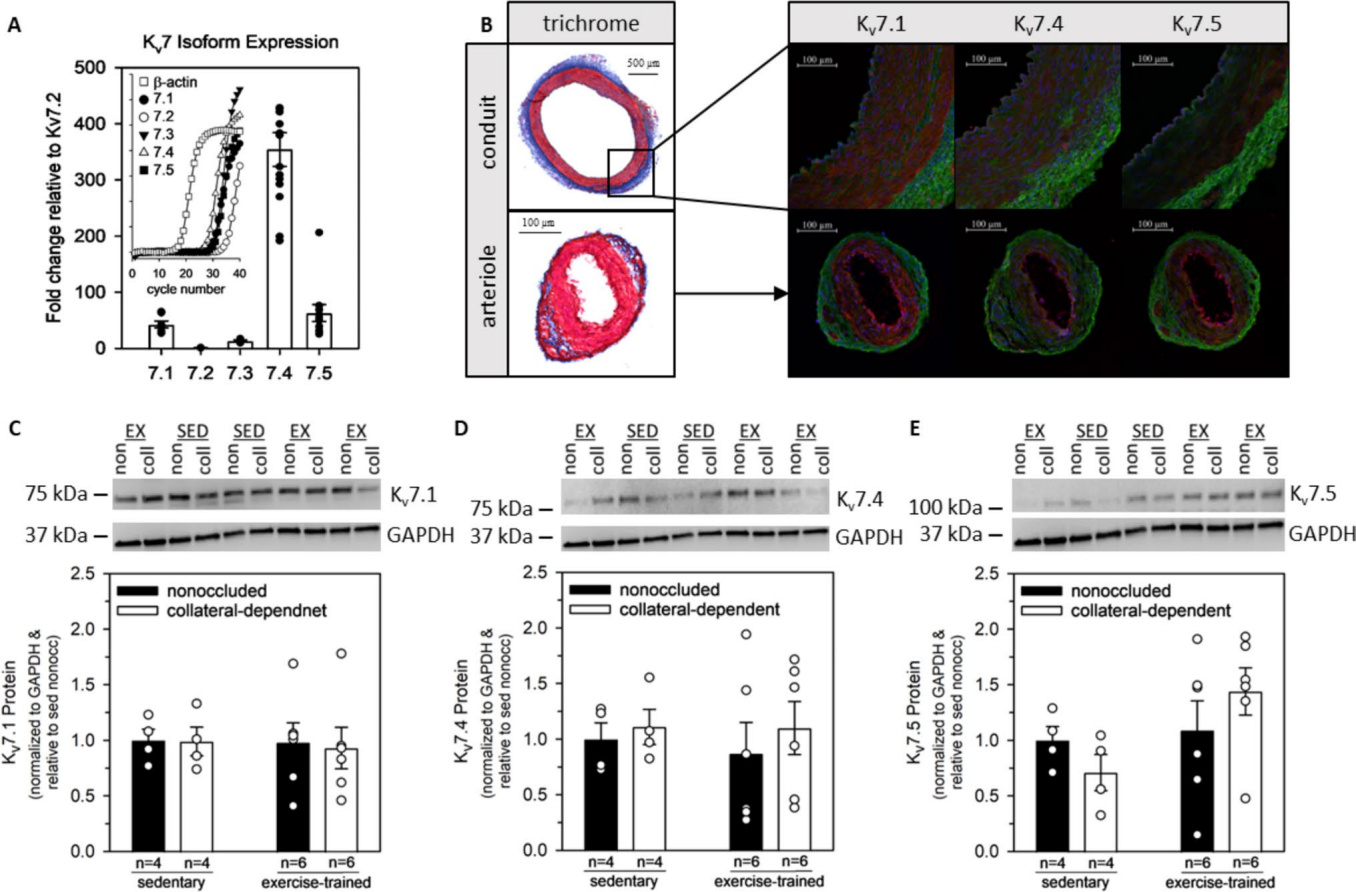
*Kv7 channel isoform expression and protein content*. **A**: Kv7 channel mRNA expression in coronary arterioles of swine is expressed relative to the housekeeping gene, β-actin, and then as a fold change relative to Kv7.2 expression, the lowest expressed gene. Our data revealed that Kv7.1, Kv7.4, and Kv7.5 were most abundant with lesser expression of Kv7.2 and Kv7.3 in porcine coronary arterioles (*n* = 4–14). **B**: Qualitative Masson’s Trichrome and immunofluorescent images demonstrating the vascular layers and differential Kv7 channel protein expression. Immunofluorescent imaging suggests higher Kv7 protein expression in coronary arterioles compared to conduit arteries. **C**-**E**: Group data (*n* = 4–6) for Kv7.1, Kv7.4, and Kv7.5 protein content (*p* = 0.990, *p* = 0.880, and *p* = 0.186, respectively). No significant changes were observed as a result of ischemia and/or exercise training. Values represented as means ± SEM

Five known isoforms of Kv7 channels (Kv7.1–7.5) are expressed throughout the body, encoded by KCNQ genes (KCNQ1-KCNQ5). We performed qRT-PCR analyses to determine the expression of KCNQ genes in coronary arterioles, relative to the reference gene β-actin (Fig. 5A). Expressions of all five Kv7 isoforms were detected with Kv7.1, Kv7.4, and Kv7.5 being the most highly expressed in whole vessel lysates of isolated coronary arterioles of abattoir swine. Immunofluorescent imaging of both conduit arteries and arterioles revealed a differential expression of Kv7 proteins (Fig. 5B). Kv7.1 was most prominent in both conduit arteries and arterioles while Kv7.4 and Kv7.5 appeared to be more abundant in arterioles compared to conduit sections. Further, both Kv7.4 and Kv7.5 fluorescence appeared most prevalent in the endothelial layer of coronary arterioles compared with smooth muscle whereas Kv7.1 protein was detected in both tissue types. Next, we examined the protein content of the most highly expressed Kv7 isoforms in our porcine model of chronic coronary occlusion and exercise training. We observed no statistical differences across all treatment groups for Kv7.1, Kv7.4, and Kv7.5 proteins (Fig. 5C-E; *p* = 0.990, *p* = 0.880, and *p* = 0.186, respectively). Immunoblot data are expressed relative to sedentary, nonoccluded arterioles.

### Contribution of Kv7 channels to whole-cell Kv current

**Fig. 6:**
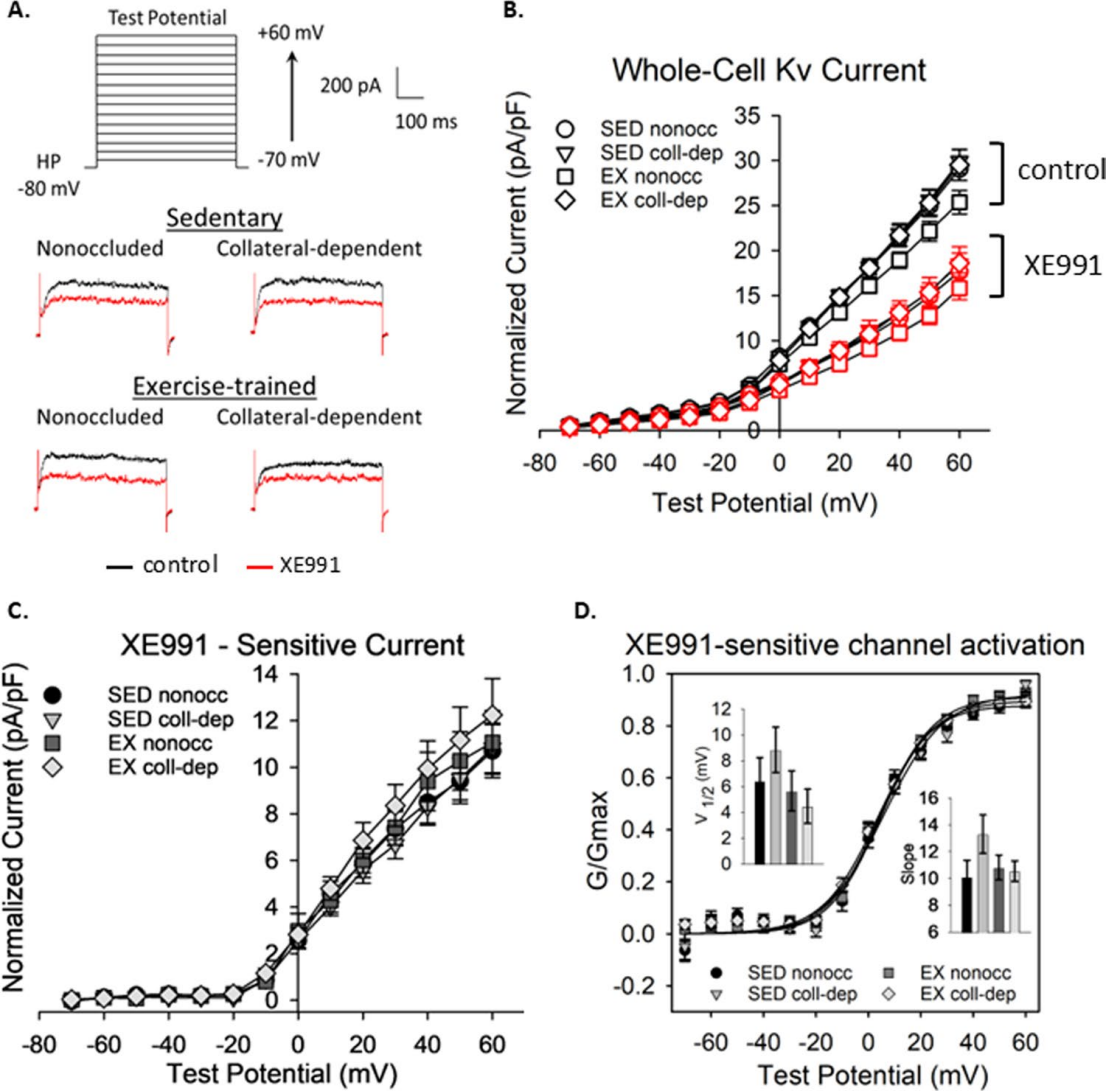
*Whole-cell Kv channel current density*. Effect of chronic coronary occlusion and exercise training on whole-cell Kv channel currents in isolated vascular smooth muscle cells. **A**: Experimental protocol (top); representative current traces at + 60 mV test potential under control conditions (black lines) and in the presence of XE991 (10 µM, red lines). **B**: Whole-cell Kv currents in the absence and presence of XE991 (10 µM). No significant effects of occlusion (*p* = 1.000 for nonoccluded v. collateral-dependent) in either sedentary or exercise-trained (*p* = 0.515 and *p* = 1.000 for sedentary vs. exercise-trained) in either nonoccluded or collateral-dependent regions were observed. XE991 significantly reduced whole-cell Kv currents in all treatment groups: sedentary nonoccluded (*p* < 0.001), sedentary collateral-dependent (p < 0.001), exercise-trained nonoccluded (p < 0.001), and exercise-trained collateral-dependent (p < 0.001). C: Whole-cell currents attributable to Kv7 channels (XE991-sensitive currents) were not different among the treatment groups (*p* = 0.943). **D**: XE991-sensitive channel activation fitted to a Boltzmann distribution with calculated V_1/2_ (inset bar graph, left) and calculated slope (inset bar graph, right). No significant differences were detected in channel activation (p = 0.686) nor sensitivity by V_1/2_ (*p* = 0.304) or slope (*p* = 0.164) across treatment groups. n = 5–8 animals, 3–4 cells/animal. Values represented as means ± SEM

To determine if impaired H_2_O_2_-mediated relaxation in collateral-dependent arterioles that was recovered by exercise training was attributable to changes in K^+^ channel activity, Kv channel currents were investigated. Figure 6A illustrates the experimental protocol with representative whole-cell current traces at + 60 mV in the absence and presence of XE991 (10 µM). No significant differences were detected between control whole-cell Kv currents as a result of chronic coronary artery occlusion (*p* = 1.000), exercise training (p = 0.515), or a combination of occlusion and exercise training (*p* = 1.000) (Fig. 6B). Cells were then superfused with 10 µM XE991, and a second set of recordings were collected. Although treatment with XE991 successfully inhibited whole-cell currents (Fig, 6B), indicated by positive XE991-sensitive currents (Fig. 6C), no significant differences were found between XE991-sensitive currents among the treatment groups (Fig. 6C; *p* = 0.943). Further, no significant differences were detected across treatment groups for the XE991-sensitive channel activation curves (*p* = 0.686), V_1/2_ (*p* = 0.304), or slope values (*p* = 0.164).

### Kv7 channel localization

**Fig. 7:**
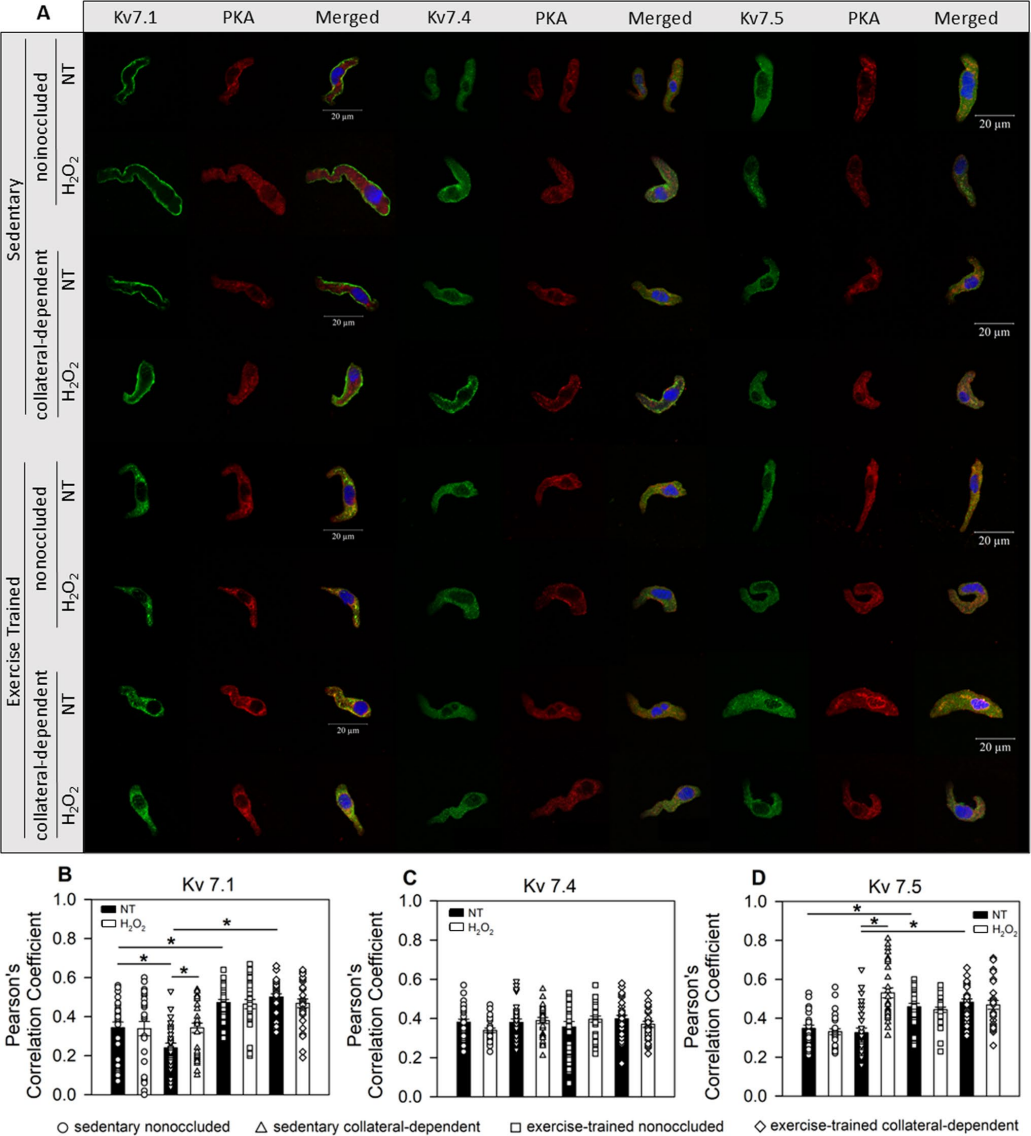
*Immunofluorescent detection of Kv7 channel isoforms and colocalization with PKA*. **A**: Representative images of Kv7.1, Kv7.4, and Kv7.5 channels (green) and PKA (red) in isolated arteriolar SMCs. **B**: Pearson’s correlation coefficient for Kv7.1 channel colocalization with PKA demonstrated a significant decrease in correlation in sedentary collateral-dependent vs. nonoccluded SMCs (*p* = 0.016) and a significantly increased correlation in exercise-trained vs. sedentary in both nonoccluded (*p* = 0.003) and collateral-dependent SMCs (*p* < 0.001). H_2_O_2_ (50 µM) treatment increased Kv7.1 and PKA colocalization only in sedentary, collateral-dependent SMCs (*p* = 0.032). **C**: Pearson’s correlation coefficient for Kv7.4 channels, demonstrated no significant differences across treatment groups. **D**: Pearson’s correlation coefficient for Kv7.5 channels revealed a significant increase in correlation as a result of exercise-training in SMCs from both nonoccluded (*p* < 0.001) and collateral-dependent arterioles (*p* < 0.001). H_2_O_2_ (50 µM) treatment increased Kv7.5 and PKA colocalization only in sedentary, collateral-dependent SMCs (*p* < 0.001). *n* = 3 animals with 10 cells/animal for all groups. *p < 0.05. Values are reported as means ± SEM

We used immunofluorescence and confocal imaging to evaluate select Kv7 channel isoform colocalization with PKA under control (non-treated, NT) and H_2_O_2_-treated (50 µM, 10 min) conditions. Representative images of Kv7.1, Kv7.4, and Kv7.5 channels (green), PKA (red), and merged images are provided in Fig. 7A. Our data reveal that PKA colocalization with Kv7.1 channels was significantly decreased in the collateral-dependent region of sedentary animals (*p* = 0.016) and significantly increased by exercise training in both nonoccluded (*p* = 0.003) and collateral-dependent arterioles (*p* < 0.001) (Fig. 7B) compared to sedentary nonoccluded arterioles. Interestingly, H_2_O_2_ treatment significantly enhanced the colocalization of PKA and Kv7.1 in SMCs of collateral-dependent arterioles of sedentary swine (Fig. 7B, p = 0.032), whereas no effect of H_2_O_2_ was observed in the other three arteriole groups. PKA colocalization with Kv7.4 channels was unchanged by chronic coronary occlusion, exercise training, or H_2_O_2_ treatment (Fig. 7C). Further, colocalization of PKA and Kv7.5 channels was not affected by chronic occlusion but significantly increased by exercise training in both nonoccluded (p < 0.001) and collateral-dependent (p < 0.001) arteriolar SMCs (Fig. 7D). H_2_O_2_ treatment also increased the colocalization of PKA and Kv7.5 in smooth muscle of collateral-dependent arterioles of sedentary swine (Fig. 7D, p < 0.001), but not in the other three arteriole groups.

## Discussion

In the present study, we investigated vascular adaptations in H_2_O_2_-mediated relaxation of the porcine coronary microcirculation in response to chronic coronary occlusion and exercise training, as H_2_O_2_ presents as a primary endothelial derived hyperpolarizing factor in coronary arterioles. In line with our previous studies [28, 60], H_2_O_2_-mediated relaxation is impaired by chronic occlusion and restored with exercise training (Fig. 2). We demonstrate for the first time that Kv7 channels contribute to the microvascular adaptations associated with IHD and exercise training (Fig. 3M–P). Specifically, we demonstrate that chronic occlusion drives a reduction in Kv7 channel contribution to H_2_O_2_-mediated relaxation that is recovered by endurance exercise training. This reduction in the role for Kv7 channels is not associated with a reduction in Kv7 channel isoform protein content by immunoblot or Kv7 (XE991-sensitive) channel activity by voltage clamp. It is noteworthy that Kv7.5 protein levels tended to be increased after exercise training and Kv7.1 tended to colocalize more with PKA after H_2_O_2_ treatment in collateral-dependent arteriolar SMCs of exercise trained compared with sedentary swine. We propose that together, these small increases in Kv7.5 protein and Kv7.1 colocalization may contribute to enhanced dilation in the collateral-dependent arterioles after exercise training or additional factors not explored play a role in the observed adaptations.

Interestingly, 4AP-sensitive Kv channels did not significantly contribute to H_2_O_2_-mediated relaxation in coronary arterioles which contrasts with previous findings demonstrating 4AP-sensitive channels are significant contributors to vascular reactivity and dependent on H_2_O_2_ signaling [45, 46, 49]. Channels in the Kv1-4 subfamilies are generally considered to be 4AP-sensitive [26, 57]. Previous studies that report a role for 4AP-sensitive channels were conducted in rodents and dogs, suggesting potential species differences. There are reports of 4AP-sensitive channels regulating coronary blood flow in vivo in a porcine model [19], but these studies do not directly link these channels to H_2_O_2_ signaling, the focus of the current studies. Additional data from the animals used in this study reveal that 4AP did attenuate whole-cell Kv currents in a manner similar to previously published reports [25] (data not shown), suggesting that 4AP-sensitive channels are present and contribute to vascular smooth muscle membrane potential in this model, but did not contribute to H_2_O_2_-mediated relaxation.

To supplement our findings, we used the Kv7 channel agonist, ML213, which is reported to be selective for primarily Kv7.4 and Kv7.5 channel isoforms, to examine the effects of direct stimulation of these isoforms on relaxation in nonoccluded and collateral-dependent arterioles of sedentary and exercise-trained swine (Fig. 4). These data revealed that arteriolar relaxation by direct activation with ML213 was significantly impaired by chronic coronary occlusion and recovered with exercise-training (Fig. 4), similar to that observed in H_2_O_2_-induced relaxation (Figs. 2 and 3M–P). Indeed, Kv7 channels are involved in the regulation of vascular tone in numerous rodent models [30, 40, 42, 54]; however, investigation of Kv7 channel contribution to the vascular reactivity of coronary microvessels with chronic ischemia and exercise training has not been reported, with the majority of studies utilizing larger coronary arteries and acute ischemic insult. Contrastingly, Goodwill et al. previously reported that Kv7 channels have no significant contribution at rest or in reactive hyperemia in a porcine model [18]. However, those studies utilized larger epicardial arteries for ex vivo analyses and acute coronary ligations (~ 15 s) as opposed to myocardial arterioles and chronic ischemic insult (~ 14 weeks). It is interesting that Kv7 channel isoforms are involved in the coronary adaptions to chronic ischemia as these channels have been reported as effective oxygen sensors in the pulmonary vasculature of rodents [50, 56]. Sensitivity to oxygen tension in vascular beds makes Kv7 channels prime candidates for regulation of blood flow during ischemic events. Although it has been reported that Kv7 channels bring about smooth muscle relaxation in response to hypoxia [23], the effects of chronic coronary occlusion on Kv7 channel activity have not been established. Our studies provide the first insight into exercise training-induced adaptations to restore the contribution of Kv7 channels in mitigating the effects of chronic coronary occlusion.

We investigated whole-cell and XE991-sensitive currents in isolated arteriolar SMCs. Whole-cell Kv channel currents were determined to be unaffected by occlusion or exercise training (Fig. 6B). Moreover, XE991 (10 µM) significantly attenuated whole-cell Kv channel currents but we detected no significant differences in the degree of inhibition as a result of occlusion or exercise (Fig. 5). However, we did observe that approximately 30% of the whole-cell current in vascular smooth muscle cells is attributable to Kv7 channels, a proportion similar to previous reports [9, 26]. These data suggest that the attenuation and recovery of Kv7 channel contribution to H_2_O_2_-mediated relaxation is not attributable to channel number or open probability in the absence of intracellular signaling axes. qRT-PCR analyses determined that all five Kv7 isoforms (Kv7.1–5) were expressed in coronary arterioles with Kv7.1, Kv7.4, and Kv7.5 being the most prominent (Fig. 5A). These results fall in line with previous studies reporting negligible expression of Kv7.2 and Kv7.3 in the coronary circulation of swine. Nonetheless, no significant differences in Kv7.1, Kv7.4, or Kv7.5 protein were detected as a result of chronic occlusion or exercise training, bolstering the hypothesis that a second messenger signaling axis is involved in the augmented H_2_O_2_-mediated relaxation response after exercise training (Fig. 5C–E).

Next, we sought to elucidate any involvement of a second messenger signaling axis that may drive these vascular responses dependent on Kv7 channel activation. Previous studies have reported that PKA associates with and activates Kv7 channels in vascular beds of multiple tissue types [4, 36, 58]. Moreover, our laboratory has previously demonstrated dimerization and subsequent activation of protein kinases by H_2_O_2_ treatment and an upregulation in PKA colocalization with BKCa channels in coronary arterioles in our porcine model of chronic coronary occlusion and exercise training [28]. Thus, we investigated the most predominantly expressed Kv7 isoforms and their colocalization with PKA in isolated SMCs from nonoccluded and collateral-dependent arterioles from sedentary and exercise-trained swine. We revealed a significant decrease in Kv7.1 channel colocalization with PKA in SMCs isolated from collateral-dependent arterioles of sedentary swine (Fig. 7B). This reduction was recovered, and colocalization of Kv7.1 channels and PKA was significantly increased in the collateral-dependent region after exercise training (Fig. 7B). Further, Kv7.5 channel colocalization with PKA was significantly increased following exercise training in both nonoccluded and collateral-dependent SMCs (Fig, 7D). Our data support a correlation between augmented Kv7 channel-dependent H_2_O_2_-mediated arteriolar relaxation and PKA colocalization with Kv7 channels following occlusion and exercise. These findings suggest that the decreased colocalization of PKA with Kv7.1 channel isoforms at the plasma membrane may underlie the decreased contributions of XE991-sensitive channels to H_2_O_2_-mediated relaxation, working under the assumption that PKA activation of Kv7 channels is H_2_O_2_-dependent in vivo, and the combined effort of PKA colocalizing with Kv7.1 and Kv7.5 isoforms is responsible for the recovered H_2_O_2_-mediated relaxation following exercise training in collateral-dependent arterioles.

It is interesting that the colocalization of PKA with Kv7.1 and Kv7.5 channels was significantly increased in response to H_2_O_2_ treatment only in the collateral-dependent arterioles of sedentary swine. We have reported previously that H_2_O_2_ levels in coronary arterioles are increased after exercise training, and that H_2_O_2_ levels were not different between the nonoccluded and collateral-dependent arterioles of sedentary swine^15^. Taken together, we postulate that intermediaries that drive translocation and colocalization of PKA/Kv7.1 and PKA/Kv7.5 are available in collateral-dependent arterioles of sedentary swine and the addition of exogenous H_2_O_2_ is necessary to assemble the mediators, whereas after exercise training, the enhanced endogenous H_2_O_2_ appears to be sufficient to maintain an elevated level of colocalization of PKA with the Kv7 isoforms such that addition of exogenous H_2_O_2_ does not increase the level of colocalization any further above resting conditions. It is unclear to us why the nonoccluded arterioles of sedentary pigs did not show enhanced colocalization of PKA and Kv7 isoforms with H_2_O_2_ treatment, although potentially under control, non-exercised conditions, the activation of Kv7 channel isoforms by exogenous H_2_O_2_ is independent of PKA.

The Kv7 antagonist, XE991, is known to inhibit all 5 isoforms of the Kv7 subfamily. ML213 is commonly known as a pore targeted activator of Kv7.2 and Kv7.4 isoforms [62]; however, subsequent studies have found that ML213 can also bind and activate Kv7.3 and Kv7.5 isoforms [5, 44]. Considering the absence of Kv7.2 and 7.3 isoforms in the coronary microcirculation, this leaves Kv7.1, Kv7.4, and Kv7.5 as possible candidates for H_2_O_2_-stimulated relaxation and Kv7.4 and Kv7.5 channels as the candidates for the response to ML213 in myography experiments. It is unknown if H_2_O_2_-mediated relaxation is dependent on an intermediary for signal transduction to activate Kv7 channels or if H_2_O_2_ directly stimulates Kv7 channel isoform through interaction with redox-sensitive residues. However, the decrease in colocalization of Kv7.1 channels and PKA in sedentary collateral-dependent arteriolar SMCs correlates with a reduced contribution of Kv7 channels to H_2_O_2_-mediated relaxation that is reversed with exercise training. It has been shown that H_2_O_2_-dependent activation of PKA by disulfide bond formation is associated with PKA mobility. Brennan et al. demonstrated the importance of H_2_O_2_-dependent disulfide formation for subcellular relocalization of PKA, revealing that the oxidized form of PKA results in an enhanced affinity for anchoring proteins in rat myocytes [3]. This process allowed for increased myofibrillar binding and PKA translocation within the cardiac myocytes, and it is possible that this process is conserved in vascular smooth muscle. There is potential that channel trafficking may play a prominent role in the adaptations observed. In fact, the microtubule network within vascular smooth muscle has a large influence on the trafficking of Kv7 channel isoforms at the cell membrane [27]. Perhaps chronic ischemia augments the functionality of the dynein protein shown to regulate Kv7 channel trafficking and this effect is reversed by exercise training. Kv7 channel trafficking as it relates to these studies will be the focus of subsequent experiments in our swine model.

## Clinical implications

Microvascular dysfunction is heterogenous and complicates diagnosis and management of cardiovascular diseases [34, 55]. Identifying novel targets for interventional treatment will broaden the scope of therapeutic strategies for afflicted patients. Taken together, our data support the notion that Kv7 channels in the coronary microcirculation may present as novel therapeutic targets in IHD. Using the clinically relevant swine model, we demonstrate that Kv7 isoforms are expressed in the smooth muscle cells of coronary arterioles, we reveal that Kv7 channels significantly contribute to vascular adaptations in H_2_O_2_ signaling in health and disease, and we provide evidence that these adaptations appear to rely on a second messenger signaling cascade. Importantly, Kv7 channel modulators are routinely used in the clinical setting for the treatment of epilepsy, depression, and anhedonia [8, 16, 52]. Kv7 channel activators targeting the Kv7.2 and Kv7.3 isoforms are employed to hyperpolarize neural tissues, decreasing epileptic frequency and improving clinical symptoms of depression. Further, Kv7 channel isoforms have been unknowingly targeted by botanical folk medicines for generations. For example, the African shrub *Mallotus* *oppositifolius* has been traditionally used across the African and Asian continents for its anticonvulsant properties and has since been identified as an activator of Kv7 channel isoforms [10, 37]. These treatments or analogues of these therapeutics might ultimately present as effective interventional strategies for the treatment of IHD by targeting Kv7 channel isoforms in the coronary smooth muscle.

## Limitations

The connection between H_2_O_2_-mediated relaxation and PKA association with select Kv7 channel isoforms is largely based on correlation. The use of targeted PKA inhibition during isometric tension myography recordings was not completed due to the lack of reliably specific PKA inhibitors [35]. The use of selective protein kinase inhibitors would have provided direct evidence of the involvement of PKA in the H_2_O_2_ signaling axis for relaxation. Moreover, there is the potential for other protein kinase isoforms, such as PKG, to be significant players in these vascular adaptations in health and disease.

In addition, a primary limitation of the studies is the restricted electrophysiological approach. Whole-cell configuration was solely used to investigate whole-cell Kv channel currents. The perforated patch approach, leaving second messenger signaling axes intact, may illuminate alterations in Kv channel activity in future studies. Utilizing the perforated patch approach, Kv currents stimulated by H_2_O_2_ or forskolin treatment may be measured and targeted by selective inhibitors. However, the electrophysiology approach regardless of configuration must be considered carefully as the vascular adaptations in response to exercise could hold an inherent temporal characteristic, such that the adaptations are lost between the time of cardiac excision and experimental protocol initiation, several hours later.

Further, the inherent variability observed in the collateral development in response to total coronary artery occlusion likely contributed to the high variability in some of the experimental measurements. This variability in concert with the difficulty and challenges of dissecting coronary arterioles created significant limitation in the quantity of experiments that could be performed. Indeed, these studies are an expansion of previous studies reported in our laboratory that experienced the same limitations.

## Conclusions

We have previously demonstrated that H_2_O_2_ production in porcine coronary arterioles is increased following exercise training as well as enhanced H_2_O_2_ contribution to endothelium-dependent arteriolar dilation [60]. In addition, we have shown that chronic coronary occlusion impairs H_2_O_2_-dependent dilation in coronary arterioles that is restored with exercise training [28]. These adaptations are mediated, in part, by augmented BKCa channel stimulation by PKA. Building on these reports, the current studies evaluated candidate Kv channel subfamilies and revealed that Kv7 channel isoforms are uncoupled from H_2_O_2_ signaling in the porcine microcirculation distal to chronic occlusion. This impairment is recovered by exercise training and attributable to restored Kv7 channel recruitment. Further, PKA association with Kv7 channels is enhanced following exercise training and may be responsible for augmented channel activity. Indeed, Kv7 channel protein appears unchanged by ischemia and exercise training, although Kv7.5 protein demonstrated trending values that could explain the loss and restoration of Kv7 contribution to H_2_O_2_ signaling. We speculate that Kv7 channel trafficking may play a significant role in these vascular adaptations, potentially being removed from the plasma membrane and internalized in response to chronic ischemia, only to be replaced following repeated bouts of exercise. Overall, these studies highlight the Kv7 channel subfamily, particularly the Kv7.1, Kv7.4, and Kv7.5 isoforms, and their modulation in a porcine model of IHD with exercise training. These exciting findings support a significant role for Kv7 channels in the development of IHD and position them as potential therapeutic targets.

## Supplementary Information

Below is the link to the electronic supplementary material.

Supplementary file 1.

## Data Availability

The datasets generated and/or analyzed during the present study are available from the corresponding author upon reasonable request.
