# Supplementary figures and images for: H2O2-mediated relaxation in a swine model of ischemic heart disease and exercise training: mechanistic insights and the role of Kv7 channels
Source: Basic Res Cardiol. 2025 Jul 12;120(5):855–72. doi: 10.1007/s00395-025-01129-6 (PMC12518372; doi:10.1007/s00395-025-01129-6)

**SUPPLEMENTARY**

Uncut immunoblot images for Kv7.1, Kv7.4, Kv7.5, and GAPDH proteins.

Kv7.1:


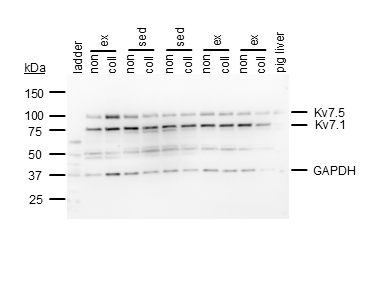


Kv7.4:


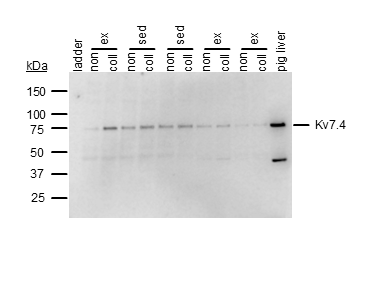


Kv7.5:


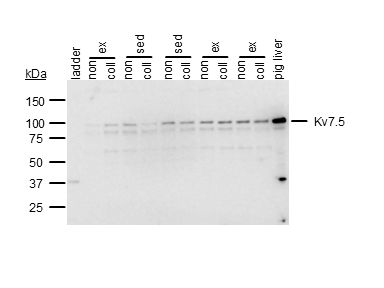

Supplement: Supplementary file 1 [file 395_2025_1129_MOESM1_ESM.docx]
